# Primary borderline retroperitoneal mucinous cystic neoplasm and situs inversus totalis: a case report

**DOI:** 10.1093/jscr/rjag918

**Published:** 2026-10-14

**Authors:** Jeude M S Bravo, Igor V Cangussu, Priscila T Tanuri, Giovanna A Rosa, Vaine G S Ribeiro, João Lucas G Correa, Lúcio Fernando D Cachaca, Frederico Augustus M Resende, Eliel O De Araújo

**Affiliations:** Department of General Surgery, Hospital e Maternidade Therezinha de Jesus, Rua Dr. Dirceu de Andrade, 33, São Mateus, Juiz de Fora, Minas Gerais, CEP 36025-330, Brazil; Faculdade de Ciências Médicas e da Saúde de Juiz de Fora – SUPREMA, Alameda Salvaterra, 200, Salvaterra, Juiz de Fora, Minas Gerais, CEP 36033-003, Brazil; Department of General Surgery, Hospital e Maternidade Therezinha de Jesus, Rua Dr. Dirceu de Andrade, 33, São Mateus, Juiz de Fora, Minas Gerais, CEP 36025-330, Brazil; Department of General Surgery, Hospital e Maternidade Therezinha de Jesus, Rua Dr. Dirceu de Andrade, 33, São Mateus, Juiz de Fora, Minas Gerais, CEP 36025-330, Brazil; Department of General Surgery, Hospital e Maternidade Therezinha de Jesus, Rua Dr. Dirceu de Andrade, 33, São Mateus, Juiz de Fora, Minas Gerais, CEP 36025-330, Brazil; Department of General Surgery, Hospital e Maternidade Therezinha de Jesus, Rua Dr. Dirceu de Andrade, 33, São Mateus, Juiz de Fora, Minas Gerais, CEP 36025-330, Brazil; Department of General Surgery, Hospital e Maternidade Therezinha de Jesus, Rua Dr. Dirceu de Andrade, 33, São Mateus, Juiz de Fora, Minas Gerais, CEP 36025-330, Brazil; Department of General Surgery, Hospital e Maternidade Therezinha de Jesus, Rua Dr. Dirceu de Andrade, 33, São Mateus, Juiz de Fora, Minas Gerais, CEP 36025-330, Brazil; Faculdade de Ciências Médicas e da Saúde de Juiz de Fora – SUPREMA, Alameda Salvaterra, 200, Salvaterra, Juiz de Fora, Minas Gerais, CEP 36033-003, Brazil; Department of General Surgery, Hospital e Maternidade Therezinha de Jesus, Rua Dr. Dirceu de Andrade, 33, São Mateus, Juiz de Fora, Minas Gerais, CEP 36025-330, Brazil; Department of General Surgery, Hospital e Maternidade Therezinha de Jesus, Rua Dr. Dirceu de Andrade, 33, São Mateus, Juiz de Fora, Minas Gerais, CEP 36025-330, Brazil

**Keywords:** retroperitoneal neoplasms, cystadenoma, mucinous, neoplasm grading, situs inversus, case reports

## Abstract

Primary retroperitoneal mucinous cystic neoplasms (PRMCNs) are rare tumors classified into cystadenoma, PRMCN with borderline malignancy (PRMCN-BM), and cystadenocarcinoma. We report a 60-year-old woman with left flank abdominal pain whose MRI revealed situs inversus totalis alongside a complex cystic lesion in the left retroperitoneum measuring 4.8 × 5.2 × 4.5 cm. She underwent laparotomy with complete resection of the lesion, and histopathology confirmed a primary retroperitoneal neoplasm with borderline malignancy, with an uneventful postoperative course and no recurrence after 8 months. To date, only 35 cases of mucinous cystadenomas with borderline malignancy have been documented in the literature. Clinical presentation is variable, imaging lacks specific diagnostic criteria, and serum tumor markers have no diagnostic value. Surgical resection remains the curative therapeutic approach, and histopathology is essential for the definitive diagnosis, which continues to pose a preoperative challenge.

## Introduction

Primary retroperitoneal mucinous cystic neoplasms (PRMCNs) are rare tumors that occur predominantly in women, and their borderline-malignancy subtype (PRMCN-BM) poses a therapeutic challenge due to its rarity. To date, ~35 cases have been described worldwide, 29 of them in women, with a mean age of 42.4 years. We present what we believe to be the 36th case reported worldwide and the second documented in Brazil, described in accordance with the revised 2025 SCARE guidelines.

## Case report

A 60-year-old woman with controlled hypertension and a history of four cesarean deliveries (the last 22 years earlier) presented with progressive left iliac fossa pain and a sensation of heaviness over 3 months, without other symptoms or relevant history. Physical examination was entirely normal, with no palpable masses and normal percussion and auscultation. The clinical timeline is summarized in Table 1.

**Table 1:** Clinical timeline

**Event**	**Date**
Preoperative consultation	3 April 2024
Elective hospital admission	24 November 2024
Surgery performed	26 November 2024
Discharge	29 November 2024
Postoperative follow-up period	3 December 2024–22 July 2025

MRI revealed situs inversus totalis (SIT) together with a complex cystic lesion in the left retroperitoneum measuring 4.8 × 5.2 × 4.5 cm, with internal septa and a peripheral nodular component, without contrast enhancement. Computed tomography (CT) confirmed the SIT and the cystic lesion, in close contact with the ascending colon/cecum, without lymphadenopathy or free fluid (Figs 1 and 2). Findings suggested a retroperitoneal mucinous cystadenoma. Serum tumor markers were not requested. Surgery was indicated.

**Figure 1:**
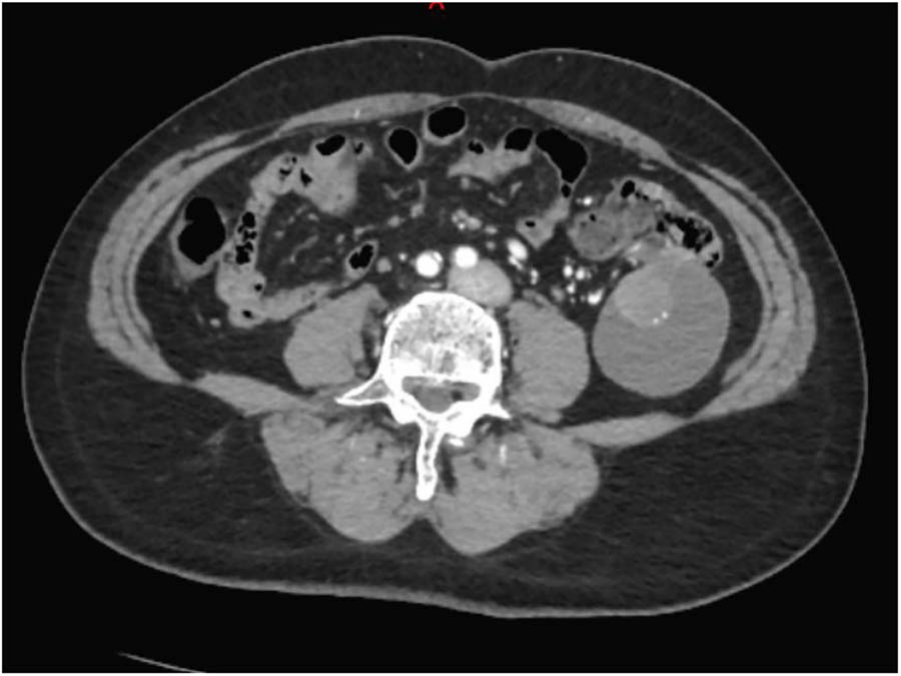
CT scan of the abdomen: shows a cystic image with regular walls, without mural nodules or vegetation, with close contact with the ascending colon/cecum.

**Figure 2:**
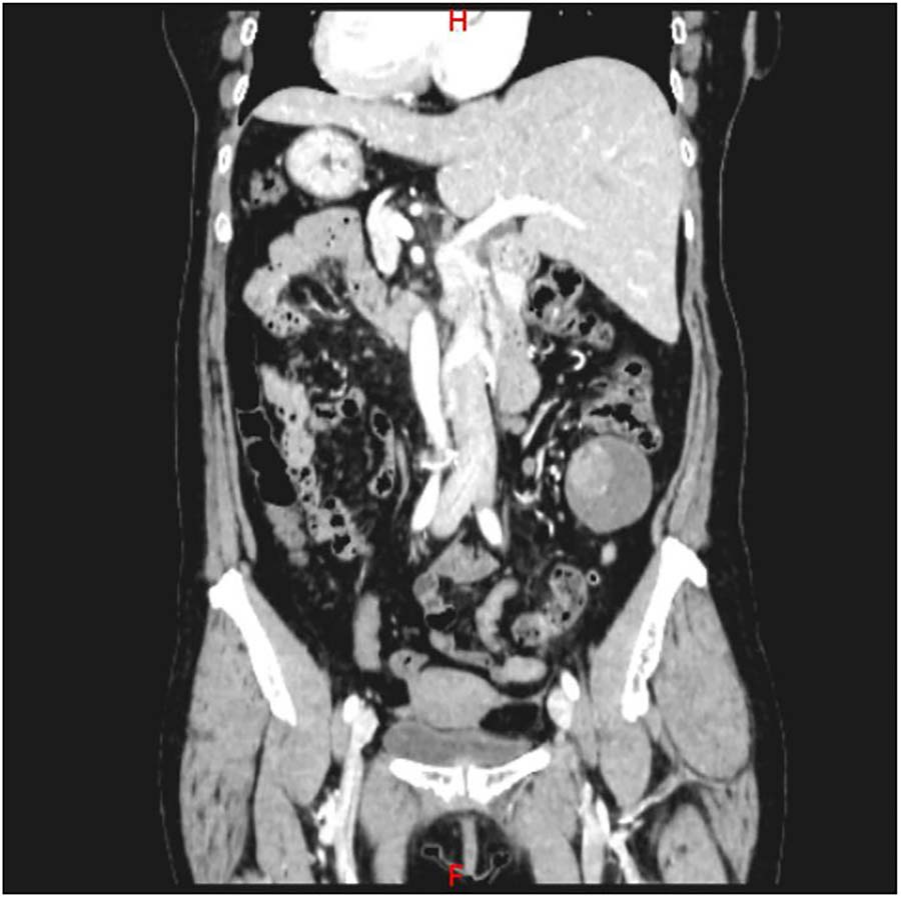
CT scan of the abdomen: shows a massive retroperitoneal cystic lesion between the left flank and the left iliac fossa.

An exploratory median laparotomy was performed under general anesthesia, led by a specialist in digestive system surgery. The retroperitoneal cystic mass, adherent to the ascending colon, was completely resected with an intact capsule (6.1 × 5.2 × 4.5 cm; Fig. 3), without rupture or spillage. An incidental appendectomy was performed due to the anomalous position of the appendix. No perioperative complications occurred. Histopathology confirmed a PRMCN with borderline malignancy, with goblet mucus-secreting cells and minimal atypia; immunohistochemistry showed expression of cytokeratins 7 and 20, CDX2, and estrogen receptor in ovarian-pattern stroma (Fig. 4).

**Figure 3:**
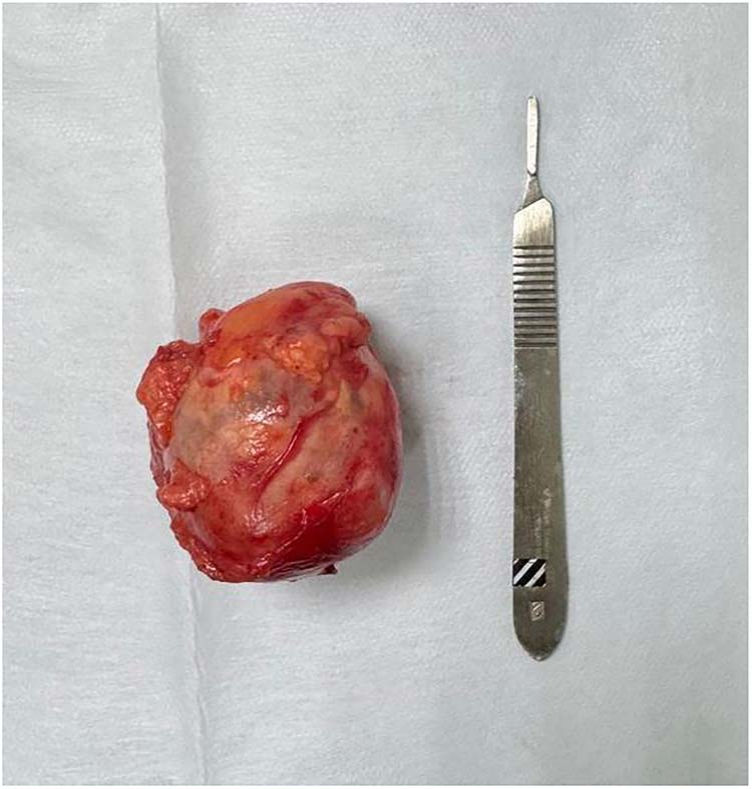
Surgical specimen: cystic lesion completely and totally resected.

**Figure 4:**
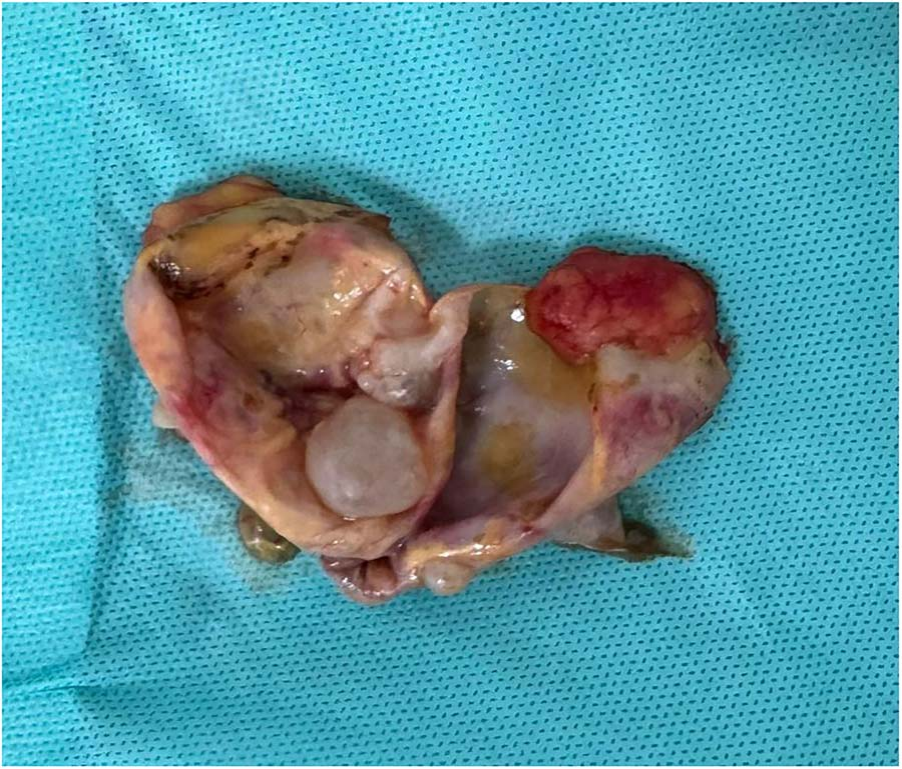
Surgical specimen: sectioned cystic lesion.

The patient was discharged on postoperative Day 3 and remains under outpatient follow-up, with no recurrence after 8 months and no indication for adjuvant therapy.

## Discussion

PRMCNs are extremely rare tumors, accounting for 0.01%–0.2% of all neoplasms, histologically resembling ovarian mucinous tumors and classified into cystadenoma, PRMCN-BM, and cystadenocarcinoma [1, 2]. To date, only 35 cases of PRMCN-BM have been reported, 29 in women, with a mean age of 42.4 years, making it a relevant differential diagnosis in young women with retroperitoneal masses [3]. Its origin remains uncertain; hypotheses include ectopic ovarian tissue, embryonic urogenital remnants, or, most accepted, mucinous metaplasia of invaginated peritoneal mesothelium [4].

Clinical presentation is nonspecific and variable, ranging from incidental findings to abdominal pain, fatigue, or palpable masses [2]. There are no specific serum markers (CEA, CA 125, CA 19-9) or pathognomonic radiological findings, making preoperative diagnosis particularly challenging [2]. Tumors range from 3 to 28 cm, usually unilocular or multilocular, rarely with a solid component [2, 5], located almost exclusively in the lateral retroperitoneal spaces.

Complete surgical resection, open or minimally invasive, avoiding spillage of mucinous fluid, is the standard curative approach [4, 6–8]. Definitive diagnosis depends on histopathology [9], and no standardized follow-up protocol exists. According to the largest available meta-analysis [10], 5-year survival is 100% for cystadenomas and borderline tumors after complete resection, versus 68% for mucinous carcinomas, giving adjuvant therapy a marginal role in borderline cases [6, 9, 10].

SIT is itself a rare congenital condition, with an estimated prevalence of ~1 in 10 000 births and a typically autosomal recessive inheritance pattern [11]. Laterality is established during gastrulation through the Nodal-PITX2 signaling cascade, the same early embryonic window in which the coelomic mesothelium differentiates; whether this temporal overlap bears any relation to the mesothelial metaplasia hypothesized to give rise to PRMCN-BM remains speculative, and no mechanistic link between the two conditions has been established [4, 11]. Beyond this pathogenetic question, SIT carries direct practical relevance for surgical planning, since mirror-image vascular and visceral anatomy can complicate both imaging interpretation and intraoperative orientation [11]. To our knowledge, no prior report has described PRMCN-BM occurring in a patient with SIT, and awareness of this possible coexistence may aid recognition and operative planning in future cases.

This report is notable for the rarity of PRMCN-BM and its unprecedented association with SIT. Limitations include the preoperative diagnostic difficulty, the absence of specific serum markers, and the lack of standardized follow-up protocols. Nonetheless, the diagnosis was clearly established, surgery proceeded without complications, and 8 months of recurrence-free follow-up confirm the success of management.

## Conclusion

Preoperative diagnosis of PRMCN-BM remains challenging and should be considered in the differential of ovarian mucinous tumors in women with normal ovaries. Early diagnosis and complete surgical resection are the mainstays of treatment, and reporting further rare cases may help establish more consistent management protocols.

## Data Availability

The datasets generated and/or analyzed during the current study are not publicly available because the data were obtained from the hospital’s internal computer-based system. The data are available from the corresponding author upon reasonable request.
